# Everolimus and temsirolimus are not the same second-line in metastatic renal cell carcinoma: a systematic review and meta-analysis

**DOI:** 10.1186/s12962-023-00420-4

**Published:** 2023-01-26

**Authors:** Zahra Goudarzi, Mehrdad Mostafavi, Mahmood Salesi, Mojtaba Jafari, Iman Mirian, Amir Hashemi Meshkini, Khosro Keshavarz, Younes Ghasemi

**Affiliations:** 1 Health Human Resources Research Center, Department of Health Economics, School of Health Management and Information Sciences, Shiraz University of Medical Sciences, Shiraz, Iran; 2 Faculty of Pharmacy, Shiraz University of Medical Sciences, Shiraz, Iran; 3 Chemical Injuries Research Center, Systems Biology and Poisonings Institute, Baqiyatallah University of Medical Sciences, Tehran, Iran; 4 Department of Public Health, School of Health, Hormozgan University of Medical Sciences, Bandar Abbas, Iran; 5 Department of Pharmacoeconomics and Pharmaceutical Administration, Tehran University of Medical Sciences, Tehran, Iran; 6 Emergency Medicine Research Center, Shiraz University of Medical Sciences, Shiraz, Iran; 7 Pharmaceutical Science Research Center, Shiraz University of Medical Sciences, Shiraz, Iran

**Keywords:** Everolimus, mRCC, Temsirolimus, Clinical trial, Efficacy, Survival

## Abstract

**Objective:**

Renal cell carcinoma (RCC) is the most common type of kidney cancer. VEGF inhibitors and mTORs are the most common therapeutic options among the different classes of available treatments. In this study, the effectiveness of Everolimus was compared to Temsirolimus, and Everolimus plusLenvatinib in renal cell carcinoma patients by review of the international clinical evidence.

**Materials and methods:**

A systematic review was conducted and all relevant published clinical studies on the efficacy and cost-effectiveness of Everolimus, Temsirolimus, and Lenvatinib plus Everolimus were searched comprehensively in electronic databases including Pubmed, Scopus, Medline, Cochrane Library, and ISI web of science. The Q score and I2 test checked the Heterogeneity and publication bias test, respectively. Egger’s test and Begg’s test were used to checking publication bias. The hazard ratio (HR) of included studies and subclass analysis were estimated by fixed and random effect models.

**Results:**

Out of 1816 found studies, ultimately, were included considering inclusion and exclusion criteria. None of these studies evaluated all three treatment strategies together and each study was about one strategy. Only one study was found for Everolimus plus Lenvatinib, so it was excluded from meta-analysis. Overall, data from 526 patients on Temsirolimus and 648 patients on Everolimus were included in Meta-Analysis. Accordingly, the efficacy of Everolimus and Temsirolimus was not statistically significant in assessed outcomes (PFS, TTSF, and death). However, Everlimus is superior to Temsirolimus in OS (Q = 3.61, p-value: 0.462, I2 = 0%). No heterogeneity or bias was detected.

**Conclusion:**

According to the results of this study, Everolimus could be related to an increase of OS versus Temsirolimus as a second line treatment of ORCC patients.

## Introduction

Noncommunicable diseases (NCDs) are now responsible for the majority of global deaths, while cancer is expected to rank the leading cause of death. Today, cancer is becoming a robust barrier to increasing life expectancy worldwide. The WHO estimates that in 2019 cancer is the first or second leading cause of death before age 70 in 112 out of 183 countries, ranking third or fourth in an additional 23 countries [1]. According to the GLOBOCAN estimation, there will be 18.1 million new cases 19.3 million new cancer cases (18.1 million excluding nonmelanoma skin cancer, NMSC) and almost 10.0 million cancer deaths (9.9 million excluding nonmelanoma skin cancer) worldwide in 2020. It is estimated that one-half of cancer cases and 58.3% of cancer deaths will occur in Asia in 2020 [1]. Among all types of cancer, urologic cancers, including bladder, prostate, and kidney cancers, are more likely to affect older individuals and males and are variably impacted by modifiable behavioral, metabolic, and environmental risk factors [2]. Kidney cancer was the seventh most common malignancy and accounted for 3.3% of all newly diagnosed cancers in 2012. Renal cell carcinoma (RCC) constitutes approximately 90–95% of all kidney neoplasms, while 25–30% of all patients had metastatic disease upon its diagnosis. The estimated economic burden of metastatic RCC was $1.6 billion (2006 USD) in selected countries. It is a rapidly evolving area of solid tumor oncology [3–5]. Cancer is the second largest group of chronic noncommunicable diseases and the third most common cause of death after cardiovascular diseases and other natural phenomena in Iran. The age-standardized incidence rates (ASIR) of cancers were 110 and 98 per 100,000 among males and females, respectively. In addition, the estimated mortality rates for cancers were 65 and 41.1 per 100,000 for males and females, respectively [6]. A meta-analysis in 2018 evaluated the incidence rate of renal cancer in Iran. The results demonstrated the low incidence rate among Iranian men (ASIR = 1.94 per 100,000); the incidence rate among Iranian women was even lower than men (ASIR = 1.36 per 100,000). According to the study results, the highest ASIR of renal cancer among Iranian men is observed in Fars (3.81 per 100,000), and the highest ASIR among Iranian women occurs in Ardabil province (2.9 per 100,000) [7].Over the past decade, medical treatment for renal cell carcinoma (RCC) has altered from a nonspecific immune approach (in the cytokine era) to a more specific therapy against vascular endothelial growth factor (VEGF) and currently to novel immunotherapy agents. Multiple agents including molecules against vascular endothelial growth factor (VEGF) and related receptor (VEGFR), mammalian target of rapamycin (mTOR) and several immune-checkpoint inhibitors—like CTLA-4, PD-1 or PD-L1 inhibitors—have been approved. Despite these advances, the most critical issue is the efficacy of biomarkers and the optimal combination and sequencing of agents [8–10].

The high costs associated with cancer care have created a difficult situation for patients in most countries. Surveying this situation will require examining the effectiveness, toxicity, and financial information of various treatment options [11, 12].

In 2012, Iran's economy collapsed under strain from sanctions imposed to stop Iran from violating the NPT Treaty. Sanctions have indirectly led to serious healthcare concerns, specifically cancer treatments. This is the first report to evaluate Iranian cancer healthcare conditions under international economic sanctions. The Program of Action for Cancer Therapy (imPACT) evaluated Iran’s NCCP, assessing multiple areas of cancer control. All areas of care were evaluated on a 9-point scale. Deficits were noted across the spectrum of care, with many areas scoring less than three out of nine. The assessment implies that Iran needs comprehensive policymaking in all areas of cancer care, especially cancer control and prevention and palliative care [13, 14].

The current study is due to the fact that the use of Everolimus technology in patients with renal cell carcinoma in Iran is not practical. We sought to investigate the clinical effectiveness of Everolimus, Temsirolimus and combination of Everolimus with Lenvatininb, by carrying out a systematic review and meta-analysis of all available evidence comparing those tree agent in clinical practice to demonstrate differences in clinical outcomes.

## Materials and methods

### Data resources and search strategy

Electronic databases including PubMed, Scopus, Cochrane Library, ISI Web of Science, and Medline were comprehensively searched using appropriate strategies, with the following keywords: mTOR, RCC, lenvatinib, Afinitor, Everolimus, Zortress, RAD001, Temsirolimus, Torisel, CCI-779, and rapamycin. The studies examined were published between 1991 and 2020.

### Inclusion and exclusion criteria

The inclusion criteria were randomized clinical trials (RCTs) articles comparing and evaluating the clinical effectiveness and cost-effectiveness of everolimus, temsirolimus, and everolimus in combination with lenvatinib in patients with RCC.

The exclusion criteria included animal studies, studies without control groups, observational studies, review studies, and economic studies. Also, studies not approved by the ethics committees and without obtaining informed consent from patients were excluded.

### Quality assessment

The Cochrane ROB tool was used to assess the quality of the selected articles. Studies with a high risk of biases were excluded from the meta-analysis process, while those indicating a low bias risk were approved.

### Data extraction

General characteristics of the included studies were extracted systematically. This quality assessment was performed by two authors independently. Any disagreements between authors were resolved through discussion.

### Data analysis

Articles with the same methodology and results were combined through meta-analysis to be used in economic evaluation.

To perform the meta-analysis, PICO included:

P (population): patients suffering RCC.

I (intervention): everolimus.

C (comparators): temsirolimus or everolimus with lenvatinib.

O (outcomes): mortality rate, overall survival (OS), time to treatment failure (TTF), and progression-free survival (PFS).

### Statistical analysis

#### Meta-analysis

For each clinical outcome of interest (OS, TTF, PFS, and death), HR was estimated using fixed and random effect models. For OS, the random effect was used due to the lack of heterogeneity in results. For others, fixed effects were used. The heterogeneity in studies’ results was tested using Cochran’s Q and I^2^. I^2^ values of 25%, 50%, and 75% were considered low, medium, and high heterogeneity. In cases of heterogeneity, subclass analyses were used to detect causes. Accordingly, the following variables were used in the everolimus and temsirolimus groups: publication year, age, sample size, treatment duration. Publication bias was also checked by Egger’s plot and Begg’s funnel plot, for which p < 0.1 were statistically significant. The statistical analysis was conducted using STATA2018.

## Results

### Study screening, characteristics, and quality of included studies

**Fig. 1:**
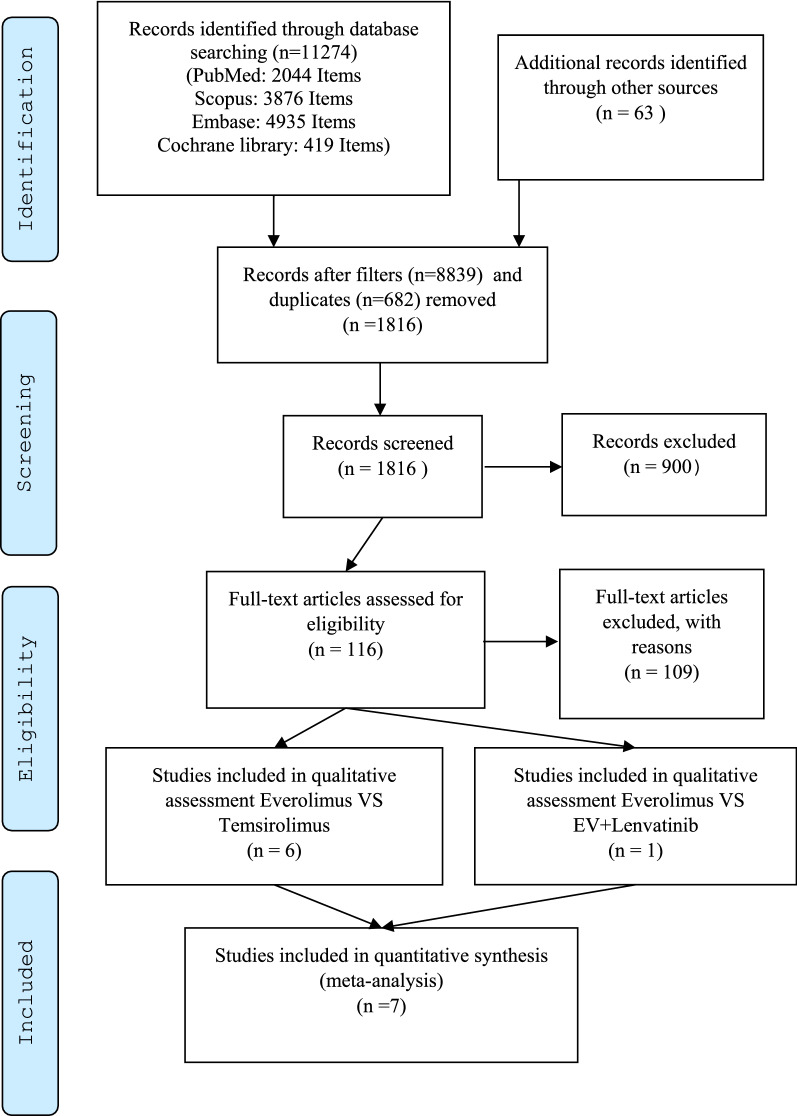
The selection process of published clinical studies for including in meta-analysis

Initially, 1816 papers were selected. Seven papers remained after screening based on the inclusion and exclusion criteria (Fig. 1). Table 1 shows a summary of the characteristics of the selected studies.

**Table 1:** Characteristics of selected studies for Meta-analysis

Study	Trial design	No. of patients	No. of patients		Intervention		Mean age (years)	
			therapy group1	Control group	therapy group	Control group	therapy	Control group
Iacovelli et al. (2014). Italy	RCT	89	65	24	Everulimus second line	Temsirolimus second line	60.3	58.2
Alimohamed et al. (2014). Canada	RCT	245	115	130	Everulimus second line	Temsirolimus second line	59	59
Wong et al. (2014). USA	RCT	401	223	178	Everulimus second line	Temsirolimus second line	64	63
Harrison et al. (2013). USA	RCT	56	19	37	Everulimus second line	Temsirolimus second line	64.3	61.8
Chen et al. (2012). USA	RCT	192	117	75	Everulimus second line	Temsirolimus second line	62	62.9
Patel et al. (2016). USA	RCT	90	59	31	Everulimus second line	Temsirolimus second line	61.6	59.6
Motzer et al. (2015). USA	RCT	101	50	51	Everulimus	Everolimus plus Lenvatinib	59	61

From the eligible trials for meta-analysis, one study was conducted in Italy, one in Canada and the others in the USA, from 2012 to 2016. No randomized controlled trial has been done so far comparing the effectiveness vs. cost-effectiveness of everolimus, temsirolimus, and everolimus combined with lenvatinib. Therefore, clinical studies related to the mentioned drugs were extracted separately and then compared. Only one study was found for everolimus plus lenvatinib; thus, it was excluded from the meta-analysis.

ROB tool was used to evaluate the quality of clinical studies; meanwhile, no study was excluded. Table 1 summarizes the search data. The quality of the studies was validated using the ROB tool. The studies that conformed to the minimum quality criteria (low risk in most domains) were included in the meta-analysis.

### Overall survival

**Fig. 2:**
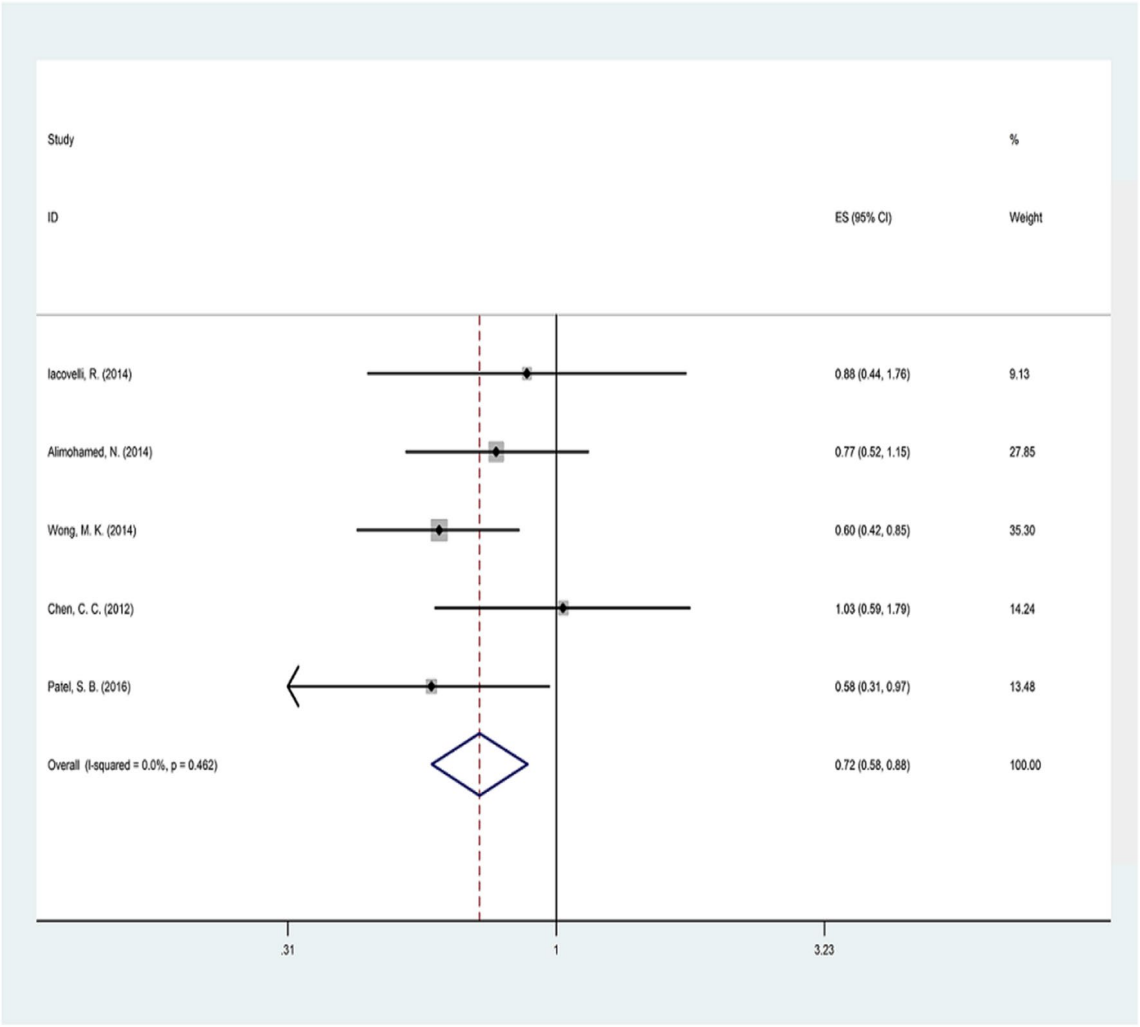
Forest plot of the selected studies regarding OS

The meta-analysis results of six studies comparing everolimus technology with temsirolimus in terms of the OS outcome are shown in Table 2 and Fig. 2. The heterogeneity test results between studies showed no significant heterogeneity (Q = 3.61, *p* = 0.462, *I2* = 0%). Consequently, using the fixed model method, the results showed that everolimus significantly reduced the risk of death by 32% compared to temsirolimus (pooled HR = 0.72, CI95% = 0.58–0.88, *p* = 0.002).

**Table 2:** Meta-analysis results

Study	Patient taking Els	Patient taking Tsls	OS			PFS			TTFS			DEATH		
			HR	CI 95%	Hetetrogeneity test	HR	CI 95%	Hetetrogeneity test	HR	CI 95%	Hetetrogeneity test	RR	CI 95%	Hetetrogeneity test
Iacovelli et al.	65	24	0.88	0.44–1.78	Q = 3.61 p-value = 0.462 I2 = 0%	0.92	0.56–1.51	Q = 15.76 p-value = 0.003 I2 = 74.6%	0.78	0.48–1.27	Q = 10.84 p-value = 0.004 I2 = 81.6%	–	–	Q = 0.74 p-value = 0.690 I2 = 0%
Alimohamed et al.	115	130	0.77	0.52–1.15		–	–		0.774	0.52–1.153		–	–	
Wong et al.	223	178	0.60	0.42–0.85		0.73	0.54–0.97		–	–		0.939	0.76–1.160	
Harrison et al.	19	37	–	–		0.54	1.13–3.61		–	–		–	–	
Chen et al.	117	75	1.03	0.59–1.79		0.48	0.30–0.79		2.05	1.26–3.35		0.716	0.398–1.288	
Patel et al.	59	31	0.58	0.31–0.97		1.03	0.63–1.69		–	–		0.78	0.224–3.425	

*ELS* everolimus, *Tsls* temsirolimus

### Progression-free survival

**Fig. 3:**
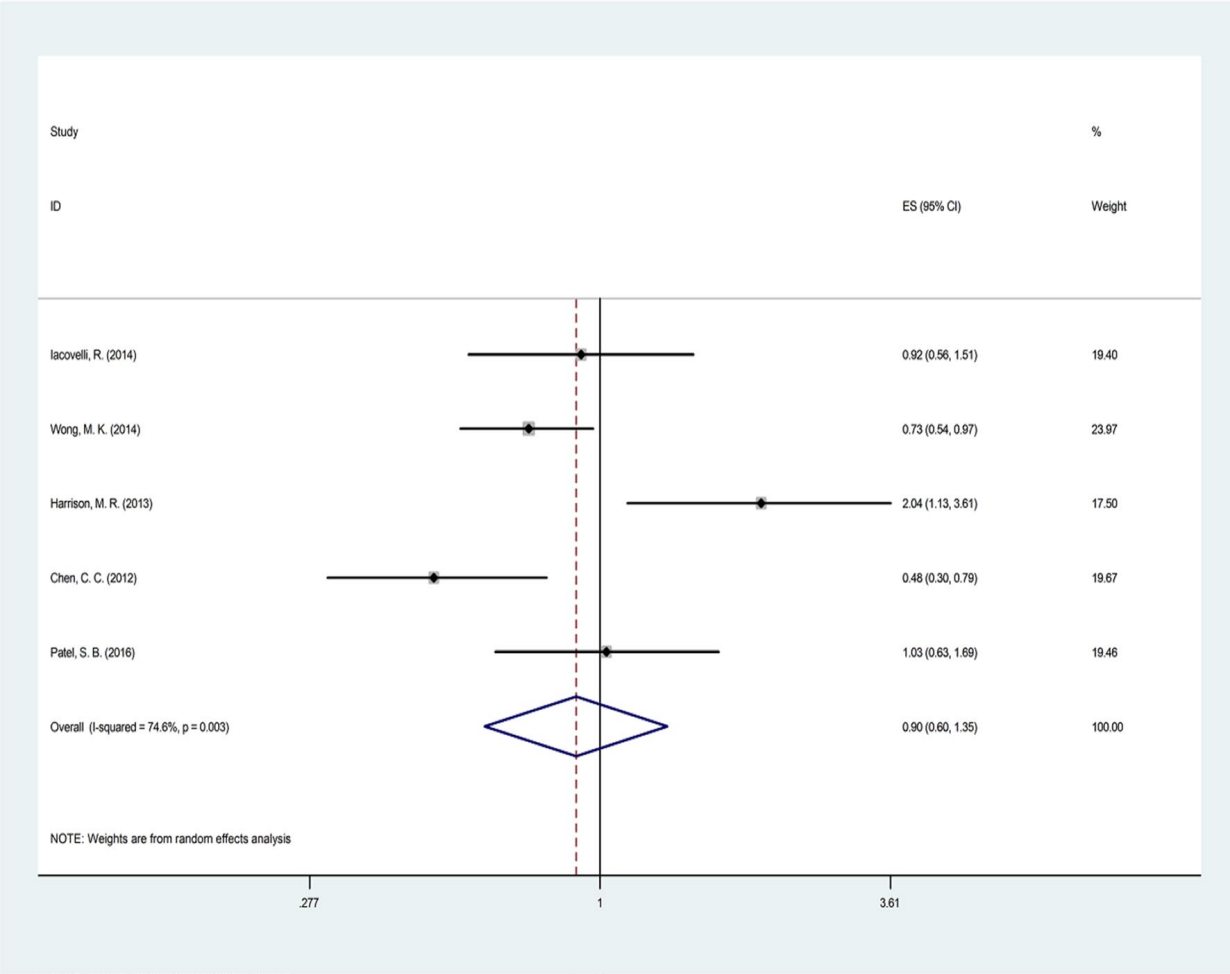
Forest plot of the selected studies regarding PFS

The results of the selected studies comparing everolimus with temsirolimus in terms of PFS outcome revealed heterogeneity results between studies (Q = 15.76, *p* = 0.003, *I2* = 74.6%) (Table 2 and Fig. 3).

The meta-regression analysis showed that none of the variables, including publication year (*p* = 0.688), total sample size (*p* = 0.338), age in everolimus group (*p* = 0.589), age in temsirolimus group (*p* = 0.631), duration of treatment in everolimus group (*p* = 0.425), and duration of treatment in temsirolimus group (*p* = 0.462), were the source of heterogeneity of studies. Hence, the random model method revealed no significant differences between patients who received everolimus and patients who received temsirolimus (pooled HR = 0.90, CI 95% 0.60–1.35, *p* = 0.608).

### Time to sequence failure

**Fig. 4:**
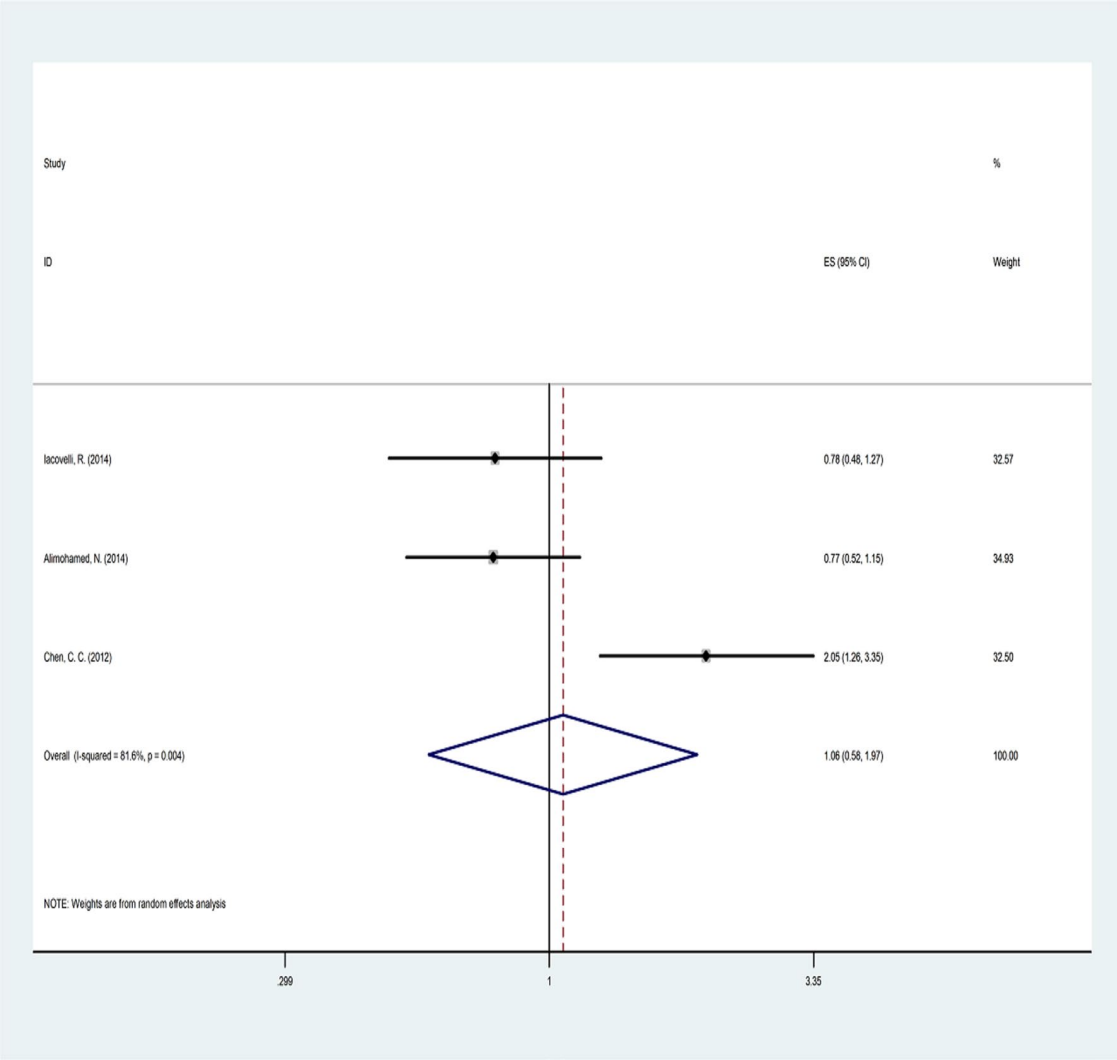
Forest plot of the selected studies regarding TTFS

The TTSF results were evaluated in only three studies on 526 patients (Table 2 and Fig. 4). As the heterogeneity of studies was reported (Q = 10.84, p = 0.004, I2 = 81.6%), the meta-regression analysis ruled out the possible variables mentioned as sources of heterogeneity. Therefore, the random model method showed no significant difference between the two technologies (pooled HR = 1.06, CI 95% 0.57–1.97, *p* = 0.841).

### Mortality rate

**Fig. 5:**
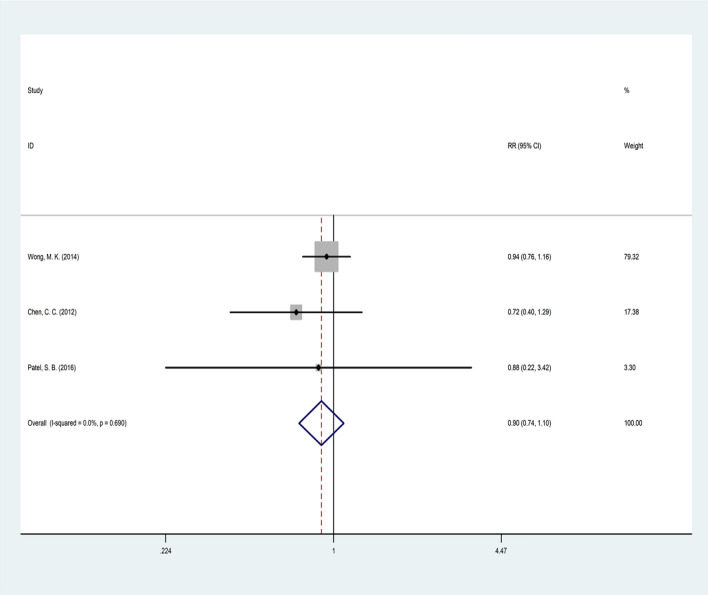
Forest plot of the selected studies regarding mortality

Three studies investigated 683 patients in terms of mortality risk by comparing two treatments (Table 2 and Fig. 5). The heterogeneity test results of studies showed homogeneity (Q = 0.74, p = 0.690, I2 = 0%). Therefore, studying the fixed model method indicated no significant differences between the efficacy of everolimus and temsirolimus (pooled HR = 0.90, CI 95% 0.74–1.09, *p* = 0.291).

### Publication bias

The visual inspection results of funnel plots and Begg and Egger's tests were used to assess the risk of bias. For all of the outcomes; OS, PFS, TTFS, and mortality, the funnel plots were symmetric, and p-values from Begg and Egger test were OS (0.327, 0.391), PFS (0.142, 0.404), TTFS (0.117, 0.615), and mortality (0.602, 0.543), respectively, no significant publication biases were detected in the study.

## Discussion

This study was the systematic review and meta-analysis on the comparative efficacy of three treatment strategies of everolimus, temsirolimus, and everolimus plus lenvatinib. Although Iacovelli et al. [15] and Heo et al. [16] evaluated temsirolimus and everolimus, a wider range of outcomes was assessed in the present study.

Thus far, no randomized controlled trials have directly compared the effectiveness vs. cost-effectiveness of everolimus, temsirolimus, and everolimus combined with lenvatinib regimen.

Studies have shown two cellular-molecular pathways for the growth of kidney cancer cells: the pathway involved with VEGF and the second pathway, the mammalian target of rapamycin (mTOR). Over the past decades, VEGF/VEGFR inhibitors including sorafenib, sunitinib, pazopanib, axitinib, cabozantinib, lenvatinib and bevacizumab, as well as two crucial mTOR pathway inhibitors, including temsirolimus and everolimus, have been identified and approved for the treatment of metastatic RCC. Unlike antiangiogenic agents, mTOR mainly acts in tumor cells, where angiogenesis-related genes involved in binding to the immunophilin FK-binding protein are inhibited. This tumor suppressor complex inhibits the activation of mTOR, which is a fundamental regulatory kinase in cell growth, cell division, motility, survival, protein synthesis, and transcription [17].

Due to the disparate pharmacokinetic properties, there have been various reports in terms of the efficacy profile of two drugs and different indications. Although temsirolimus, an intravenously administered agent, is approved based on the results of a phase III trial in the first-line therapy setting for patients in the poor-risk prognosis category, it has occasionally been used instead of everolimus [18], while everolimus is recommended for patients previously treated with at least one VEGFR-TKI [19].

Both drugs are metabolized in the liver, but temsirolimus is converted into an active metabolite, sirolimus, and has a half-life of approximately thirteen hours. Everolimus has four active metabolites; therefore, the half-life is almost two-folded. Furthermore, everolimus is partitioned into erythrocytes at a higher therapeutic concentration [20, 21].

Recent studies suggest that everolimus offers superior OS compared to temsirolimus after disease progression during VEGFR-TKI therapy for patients with mRCC, although both agents are associated with similar response rates and PFS [22]. The differences in pharmacokinetics might partly explain the difference in efficacy. The longer half-life of everolimus and that most of the drug is partitioned into erythrocytes might lead to more sustained inhibition of mTOR activity, even after discontinuation of the drug, resulting in greater efficacy in terms of OS compared with temsirolimus.

Various clinical trial studies have shown that lenvatinib combined with everolimus can exhibit antiangiogenic and anti-tumor effects by suppressing VEGFR, FGFR, and mTOR signaling pathways. A phase II clinical trial in the U.S. and EU also showed that lenvatinib at 18 mg/day and everolimus at 5 mg/day compared with 10 mg/day everolimus monotherapy has significantly improved RCC patients. Finally, this combination regimen was approved in patients with RCC after treatment with anti-angiogenesis drugs and treatment with VEGF-targeted agents [23].

Derkach et al. have reported the cost-effectiveness of the combination regimen with lenvatinib and everolimus in patients suffering RCC compared to monotherapy with nivolumab, which has reduced the costs of treatments 29.9% [24].

In another cohort study investigating 426 mRCC patients, the cost-effectiveness of everolimus and temsirolimus was compared. The results showed that regardless of whether the two drugs were used in the first, second, or third line of treatment, with the same disease stage and similar demographic characteristics, the administration of everolimus was independently associated with lower use of outpatient healthcare resources than temsirolimus [25]. Also. Ebara et al. reported that patients taking everolimus had lessened the indirect medical cost by reducing their outpatient referrals compared to temsirolimus [26].

Motzer et al. compared lenvatinib, everolimus and the combination of lenvatinib plus everolimus in a phase II clinical trial study. Results showed that lenvatinib plus everolimus significantly prolonged PFS compared to everolimus alone.

Iacovelli et al. utilized a similar methodology. They achieved similar results in agreement with our analysis results [15]. In terms of the OS outcome, four studies were included in the meta-analysis. The data of 937 patients taking everolimus and temsirolimus as second-line therapy were investigated. The everolimus regimen reduced the risk of mortality by 26%, resulting in the predominancy of everolimus over temsirolimus by decreasing the risk of treatment failure by 30%, considering the TTF were evaluated in 692 cases [14]. In the Heo et al. study, everolimus slightly improves the PFS index compared to temsirolimus, while there was no significant difference in the OS index [15].

Furthermore, in this paper, the five studies investigating 1017 patients in terms of OS consequence revealed that the everolimus regimen significantly reduced the risk of death by 32% compared to temsirolimus, making it a superior option. In contrast, there were no significant differences in comparing the effectiveness of PFS, TTSF, and death. Also, the comparison made between Everolimus and Everolimus + lenvatinib using only one available study and results revealed that the combined regimens of two drugs are more effective than the Everulimus alone, in terms of PFS index and OS.

Finally, this combination regimen was approved in patients with RCC following one prior vascular endothelial growth factor-targeted therapy. Due to insufficient studies, comparing the everolimus with combination therapy with lenvatinib did not result in accurate conclusions; thus, further clinical studies are needed on the issue.

## Conclusion

According to the results of this study, everolimus seems to be superior to temsirolimus in MRCC patients. However, additional cost-effectiveness evidence is required for more precise decision-making.

## Data Availability

All data are available on reasonable request.
